# Superlattices: problems and new opportunities, nanosolids

**DOI:** 10.1186/1556-276X-6-127

**Published:** 2011-02-10

**Authors:** Raphael Tsu

**Affiliations:** 1 University of North Carolina at Charlotte, Charlotte, NC 28223 USA

## Abstract

Superlattices were introduced 40 years ago as man-made solids to enrich the class of materials for electronic and optoelectronic applications. The field metamorphosed to quantum wells and quantum dots, with ever decreasing dimensions dictated by the technological advancements in nanometer regime. In recent years, the field has gone beyond semiconductors to metals and organic solids. Superlattice is simply a way of forming a uniform continuum for whatever purpose at hand. There are problems with doping, defect-induced random switching, and I/O involving quantum dots. However, new opportunities in component-based nanostructures may lead the field of endeavor to new heights. The all important translational symmetry of solids is relaxed and local symmetry is needed in nanosolids.

## Introduction

Of all the thousands of minerals as jewelry, only a few are suitable for electronic devices. Silicon, in more than 95% of all electronic devices, GaAs-based III-V semiconductors, in the rest of the optical and optoelectronic devices, and less than 1% used in all the rest such as lasers, capacitors, transducers, magnetic disks, and switching devices in DVD and CD disks, comprise a very limited lists of elements. For this reason, Esaki and Tsu [1,2] introduced the concept of man-made superlattices to enrich the list of semiconductors useful for electronic devices. In essence, superlattice is nothing more than a way to assemble two different materials stacked into a periodic array for the purpose of mimicking a continuum similar to the assemble of atoms and molecules into solids by nature. Although it was a very important idea, the technical world simply would not support such activity without showing some unique features [3]. We found it in the NDC, negative differential conductance, the foundation of a high speed amplifier. In retrospect, man-made superlattice offers far more as well as branching off into areas such as soft X-ray mirror [4], IR lasers [5], as well as oscillators and detectors in THz frequencies [6]. The very reason why such venture took off is because the availability of new tools such as the molecular beam epitaxy, MBE, with in situ RHEED, better diagnostic tools such as luminescence and Raman scattering, the all important TEM and SEM, etc. After the introduction of scanning tunneling microscopy, STM; and atomic force microscopy, AFM, stage is set for further extension of quantum wells, QWs, into three-dimensional structures, the quantum dots, QDs. The demand of nanometer regime is due to the requirement of phase coherency: the electrons must be able to preserve its phase coherency at least in a single period, on reaching the Brillouin zone in k-space. However, we shall see why new problems developed in reaching the nanometer regime. First of all, when the wave function is comparable to the size, approximately few nanometers in length, it is very similar to a variety of defects. Strong coupling to those defects results in random noise, the telegraph switching [7]. Thus we are facing great problems in pushing nanodevices. However, some of the new frontiers in these nanostructures are truly worthy of great efforts. For example, chemistry deals with molecules largely governed by the symmetry relationship within a molecule. In solids the symmetry is governed by the translational symmetry of unit cells. Now, with boundaries and shape to contend with, we are dealing with a new kind of chemistry, involving the symmetry of surfaces and boundaries as well as shapes. For example, we know that it is unlikely a tetrahedral-shaped QD may be constructed with individual linear molecules. Catalysis is still a matter of mystery even today. Now we are talking about adding boundaries and shape for nanochemistry. The possibility of crossing over to include biological research of nanostructures is even more spectacular, which will ultimately lead mankind into the physics of living things.

## Problems

### Response of a superlattice: DC and AC

Following [2], for a simple sinusoidal variation of potential, a very simple relationship may be obtained simply by integrating the equation of motion with a field *F *$\hbar \frac{\text{d}k_{x}}{\text{d}t}=eF$, with the expression for velocity, $v_{x}=\hbar^{-1}\frac{\partial E_{x}}{\partial k_{x}}$, we can write the current from $v_{\text{d}}=eF\hbar^{-2}\int \left(\partial^{2}E_{x}/\partial k_{x}^{2}\right)\exp\left(-t/\tau \right)\text{d}t$, in which *τ *is the collision time. Taking a sinusoidal *E *- *k *relationship, the so-called tight binding dispersion relation with a period of *d*, the drift velocity

(1) $$v_{\text{d}}=g\left(\xi \right)\left[\hbar k_{\text{d}}/m(0)\right],\;\text{where }g\left(\xi \right)=\xi /\left(1+\pi^{2}\xi^{2}\right)$$

in which ξ ≡ *eFτ*/*ħk_d_*, *m*(0) = 2*ħ*^2^/*E*_1_*d*^2^, and *k_d _*= *π*/*d *is the Brillouin zone *k*-vector. Note that at low field, small ξ, *v*_d _is ohms law. But at high field, the drift velocity goes down with field, therefore NDC, the basic requirement for amplification, which is the foundation for oscillators. Note that for large *τ*, the drift velocity goes to zero so that the steady current disappears, leaving only pure oscillation. This is the basic Bloch Oscillation. With time varying fields, at frequency ω_1_, the velocity is now

(2) 〈vx〉=〈vx〉0+Re〈vx〉1cosω1t+Im〈vx〉1sinω1t,

(3) $${\langle v_{x}\rangle }_{0}=v_{0}H\frac{\omega_{\text{B}}\tau }{{\left(\omega_{\text{B}}\tau \right)}^{2}+1},$$

and *ω*_B _≡ *eF*_0_*d*/*ħ *and *ω*_B1 _≡ *eF*_1_*d/ħ *with *F*_0 _the dc field and *F*_1 _the ac field, Equation 3 is identical, as it should be, to the previous results Equation 1, except the factor *H. H *= 1, then *v*_0_*H *= *E*_1_*d*/*ħ *and the maximum extent 〈*x*〉*_m _*= 〈v*_x_*〉*_m _τ *= *E*_1_/*2eF*_0_. The length is measured by *nd*, with *n *being the number of periods. The electrons will now oscillate with a period *T *= 2*π*/*ω*_B_, which was known to Bloch and discussed by Houston [8]. Without collision, an electron will oscillate at a frequency of *ω*_B _and cover a distance of *E*_1_/*eF*_0_. The extent of an electron without collision is twice the maximum distance given by *ω*_1_*τ *= 1. The velocity [9] is given by:

(4) $$\langle v_{x}\rangle \;=v_{0}H\sum_{m,n=-\infty }^{\infty }J_{m}\left(\frac{\omega_{\text{B}1}}{\omega_{1}}\right)J_{n}\left(\frac{\omega_{\text{B}1}}{\omega_{1}}\right)\frac{\sin(m-n)\omega_{1}t+(\omega_{\text{B}}+n\omega_{1})\tau \cos(m-n)\omega_{1}t}{{\left(\omega_{\text{B}}+n\omega_{1}\right)}^{2}\tau^{2}+1}$$

The in-phase component with time goes as cos *ω*_1_*t *which we abbreviate by writing *Re*〈*v_x_*〉 and the out-of-phase component with time goes as sin *ω*_1_*t *is abbreviated by Im〈*v_x_*〉. In linear response, we sum for *n*-*m *= 1. The equations describing the linear response are given below:

(5) Re〈v〉≡Re〈vx〉1v0cosω1t(2ωωB1) and Im〈v〉≡Im〈vx〉1v0sinω1t(2ωωB1)

**Figure 1:**
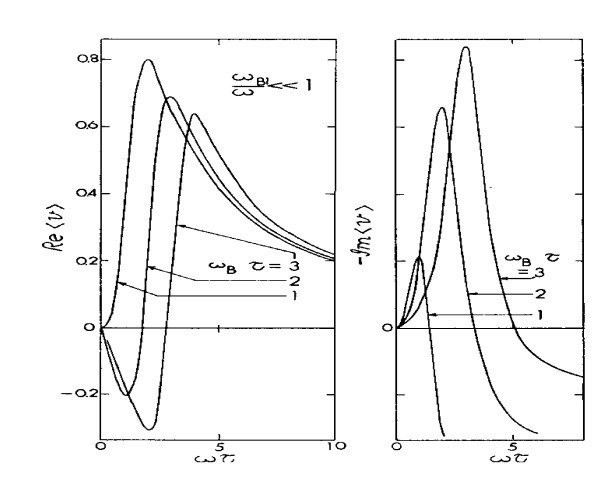
**The in-phase, *Re*〈*v*〉_1 _and out-of-phase Im〈*v*〉_1 _components of the linear response function for a superlattice with an applied electric field of *F *= *F*_0 _+ 2*F*_1_cos*ωt*, *ω*_B _= *eF*_0 _*d/ħ*, and *ω*_B1 _= *eF*_1 _*d/ħ***.

In Figure 1, for *ω*_B_τ = 1, *Re*〈*v*〉 is always positive indicating the lack of gain or self-oscillation. The Im〈*v*〉 has a maximum at *ω *= *ω*_B_. For *ω*_B_τ = 2, *Re*〈*v*〉 has a minimum at *ω *= *ω*_B_/2 and is negative, but Im〈*v*〉 has a peak at *ω *= *ω*_B_. With a further increase to *ω*_B_*τ *= 3, *Re*〈*v*〉 has a maximum negative value at *ω *= 2*ω*_B_/3 and the Im〈*v*〉 has a peak at *ω *= *ω*_B_. Thus the peak in Im〈*v*〉 always appears at *ω *= *ω*_B_, substantiating the intuitive understanding that the system is oscillating at the Bloch frequency. The question of gain or loss is another matter as we need to focus on *Re*〈*v*〉. Note that *Re*〈*v*〉 always has a maximum negative value below *ω*_B, _indicating that self-oscillation that occurs at the maximum gain is never at the Bloch frequency. Only as *ω*_B_*τ *→ *∞ does the maximum gain coincide with the Bloch frequency*. For both *ω*_B_*τ *≫1 and *ωτ *≫ 1, it is seen that *Re*〈*v*〉_3 _can have a substantial region that is negative, indicating that in the region of nonlinear optics, an intense optical field is needed for gain. What is happening is that higher energy photons cause transitions between mini-bands, providing additional nonlinear response. This is because *k *is conserved to within multiples of the reciprocal lattice vector, as in *umklapprozesse*. In the usual solids, optical nonlinearity arises from small non-parabolicity of the *E-k *relation as treated by Jha and Bloembergen [10]. However, *in man-made suprelattices, non-parabolicity is huge, leading to substantial 2nd and 3rd harmonics *[11].

### When are the full Bloch waves needed?

**Figure 2:**
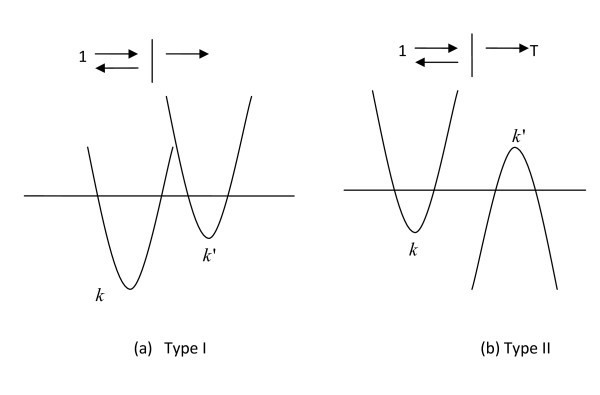
***E-k *for (a) type I and (b) type II superlattices, with energy at horizontal line**.

Figure 2a shows a type I-superlattice, i.e., an electron in a conduction band incident to the left of another conduction band separated by an interface and a type III-superlattice in (b) where the right side is a valence band at the same energy.

Explicitly [12], the superscripts (+) and (-) denote the waves moving to the right and left, respectively, and the subscripts c and v denote the conduction and valence bands, or the upper and lower bands:

(6) ψc+=Uc(k,x)eikx, ψc−=Uc(−k,x)eikxψv+=Uv(−k′,x)eik′x, ψv−=Uv(k′,x)e−ik′x

Let us proceed with the reflection problem, with an electron from the left conduction band and emerging from the right of the interface into the conduction band with (+) for *k*_2_, and valence band with (-) *k*_2_. The conduction band electron incident from the left onto an interface located at *x *= 0, we use *U*_1 _≡ *U*_c _(*k*_1_, *x*), *V*_1 _≡ *U*_c _(-*k*_1_, *x*); and for the transmitted electron to the right, *U*_2 _≡ *U*_v _($\mp {k^{\prime }}_{2}$, *x*), (-) for movement to the right and (+) for movement to the left, then

(7) $$\psi_{1}=U_{1}\exp\left(ik_{1}x\right)+R\;V_{1}\exp\left(\;-\;ik_{1}x\right),\;\text{and}\;\psi_{2}=T\;U_{2}\exp\left(ik_{2}x\right)$$

Matching these wave functions and their derivatives and for equal effective masses

(8) $$R=\frac{\left(k_{1}-k_{2}\right)-i\left[\left({U^{\prime }}_{1}/U_{1}\right)-\left({U^{\prime }}_{2}/U_{2}\right)\right]}{\left(k_{1}+k_{2}\right)-i\left[\left({U^{\prime }}_{2}/U_{2}\right)-\left({V^{\prime }}_{1}/V_{1}\right)\right]},\;\text{and}\;T=\frac{2k_{1}-i\left[\left({U^{\prime }}_{1}/U_{1}\right)-\left({V^{\prime }}_{1}/V_{1}\right)\right]}{\left(k_{1}+k_{2}\right)-i\left[\left({U^{\prime }}_{2}/U_{2}\right)-\left({V^{\prime }}_{1}/V_{1}\right)\right]}$$

Therefore, for Type III, the traditional reflection coefficient *R *and transmission coefficient *T *involve the *U *and *V *Bloch functions. In general, Bloch waves should be used. The smaller the period, the larger is the interaction resulting in coupling and larger bandgap. Therefore, Type III gives rise to bandgap by design. However, if the period > coherent length, the system returns to semi-metallic [12].

Resonant tunneling in a single quantum well with double barriers

#### Some important issues in resonant tunneling

It was pointed out by Sen [13] that time-dependent Schrodinger equation should be used dealing with the question on tunneling time. However, using the simple delay time defined by

(9) τ=dφ/dω=(dφ/dk)(dk/dω), φ=kd+θ,

where ϕ is the total phase shift and θ is the phase of the transmission amplitude through the DBRT structure. The delay time τ for a structure obtained from solving the time-dependent Schrodinger equation using Laplace transform is in fact close to the approximate values in Equation 9.

There is an important point. The computed transmission time generally oscillates during the initial time, reaching several orders of magnitude down from the delay time. If the energy is at resonance, the delay time rises and overshoots approximately 8% [14] and settles down to the delay time using Equation 9, something quite familiar with most transient analyses. In time-dependent microwave cavity with *E *&*M *waves, there is generally similar delay in time response at resonance. And at off resonance, a small fraction does get through quickly, but many orders of magnitude down. There is no need to argue about tunneling time as during 1960 s. If we really need to know, particularly with special circumstances, we should solve the time-dependent wave equation using Laplace Transform.

There were issues concerning one-step resonant tunneling through a DBQW and two-step process [12] pointed out that Luryi's two-step process is almost indistinguishable from resonant tunneling when the loss factor is fairly high, which is the case for most DBQW.

There are many issues concerning resonant tunneling. For example, some sort of intrinsic instability was suspected. However, at the end, it was resolved by recognizing that the very scheme for setting off the heavily doped contact away from the DBQW structure introduces an extra QW under bias [15].

#### Conductance from tunneling

The expression for the conductance in tunneling in terms of the transmission *T *[12]

(10) $$G=\frac{e^{2}}{h}\sum_{n,m}T\left(E_{F},n,m\right),$$

where the sum is over the transverse degree of freedom (*n, m*), or integration in d*k*_t _of the transverse channels, and $T\equiv T*T({k^{\prime }}_{l}/k_{\ell })$. The conductance per transverse channel is $G_{nm}=\frac{e^{2}}{h}T_{nm}$. If in each transverse channel *T_nm _*= 1, then $G_{nm}=G_{0}=\frac{e^{2}}{h}$, the so-called quantum conductance. This last assumption is frequently made; however, it is noted that the condition *T_nm _*= 1 gives zero reflection, which happens near resonance and is contrary to the assignment of a contact conductance. In transmission line theory the only reflection-less contact is one with the input impedance exactly equal to the characteristic impedance, a wave impedance of the line. Let us discuss more in detail. First of all, an impedance function is merely a special case of a response function or transfer function for the input/output. Therefore, there is no such thing unless two contacts are involved serving as input and output. The impedance or conductance has been referred to as contact conductance by Datta [16]. In reality it is not a contact conductance. If *T *= 1 is taken, then Equation 10 applies to reflectionless. The real issue is why experimentally equal steps of *G*_0 _appear? *I think the answer lies in the fact that all the transverse modes are not coupled with a planar boundary..*.More precisely, *G*_0 _is the conductance of the quantum wire with matched impedance at the input end and terminating in the characteristic impedance, the wave impedance for electron, therefore also matched at the output end. Letting a wave bounce between two reflectors adopted by Landauer [17] for the conductance is a special case of the general time-dependent solution [12].

#### Noise and oscillation in coupled quantum dots

**Figure 3:**
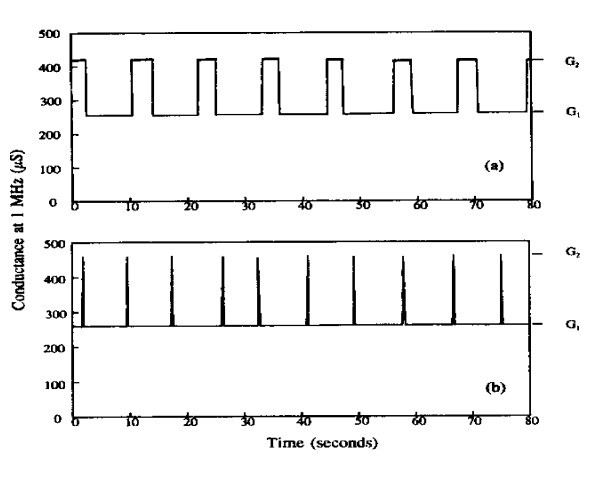
**Conductance oscillations between *G*_1 _and *G*_2 _at biases: -11.95 V**. (a) Near *V*_*n*+1_; and -11.85 V, (b) near *V_n_*, the voltage arbitrarily assigned on *G *versus bias voltage.

When many Quantum Dots are coupled under one contact, due to the good coupling between the wave functions of the QDs to the wave functions of the defects, uncontrollable oscillations referred to as telegraph noise appeared. Figure 3 shows a typical case of many Si-QDs with size approximately 3 nm. The switching speed changes from approximately 2 s to more than 10 s. Note that Δ*G *= *G*_2 _- *G*_1 _= 420 - 260 μS = 160 μS - 4*G*_0_, indicating that 4 electrons participated in the conduction process. We have observed oscillations lasting for an entire day. But, in some cases oscillation stops after only 900 s as if we have used up the QDs involved [18].

**Figure 4:**
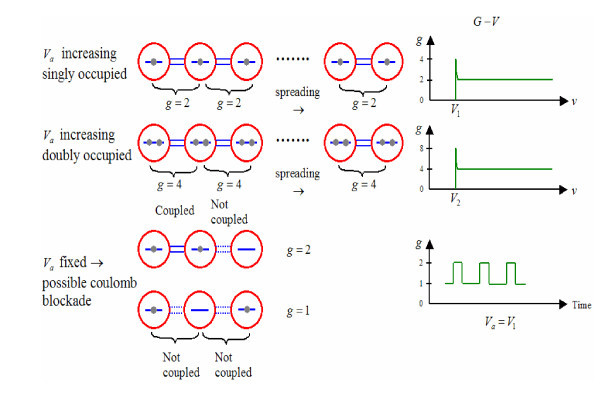
**A Model for the enhanced coupling between QDs from adjacent QDs**. Top shows singly occupied individual QDs, middle shows doubly occupied QDs, and bottom shows exchanging occupations leading to oscillations generally fast oscillations. When a trap serves as an imposter of a QD, telegraph-like slow oscillation occurs [19].

We now basically understood this telegraph-like noise. Figure 4 shows how QDs are coupled together much the same way as molecules. Whenever two adjacent QDs are occupied, the self-consistent potential moves up at the expense of the barrier separating them. This process goes on as the dots are coupled in forming two-dimensional sheets until something happens; no dots are within the coupling range. The wave function of the QDs is affected by strong coupling with that of the defects, even for defects located relatively far from the locations of the dots, strong 1/*f *noise, commonly known as telegraph noise appears. In fact this type of problem even occurs in optical properties of QDs, blinking in emission [19]. One may argue that this switching is due to very large defects of a Si matrix, these Si nanocrystals are embedded. My view is that reducing these defects is possible, but eliminating them is not possible.

#### Capacitance, dielectric constant, and doping of QDS

**Figure 5:**
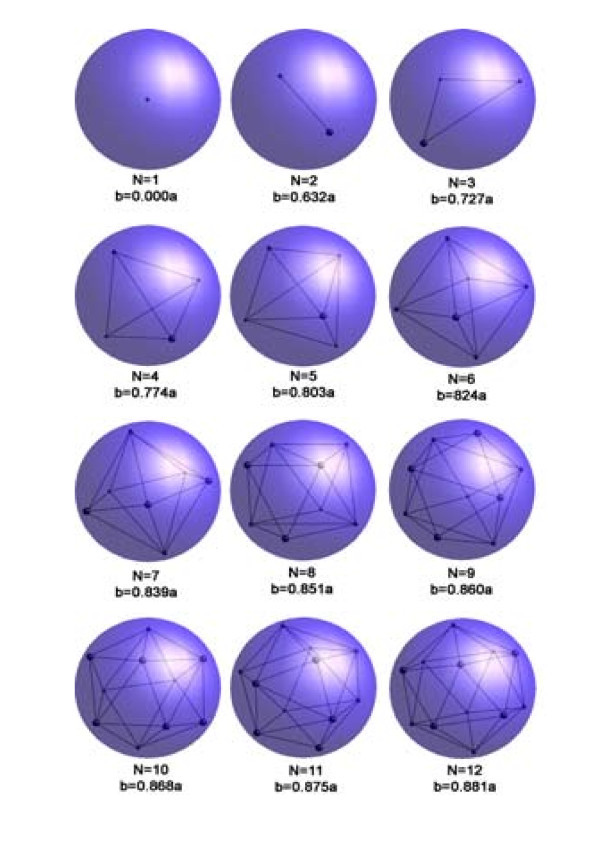
***N*-electrons in a dielectric sphere**. After Zhu et al. [29].

**Figure 6:**
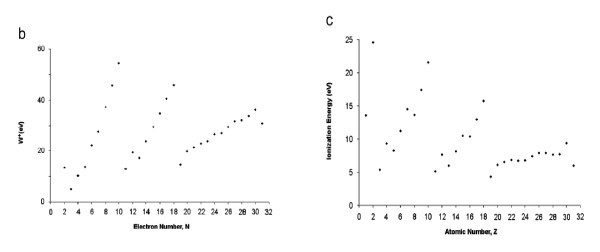
**a) Interphasic energy *W^+ ^*= *E*(*N *- 1 + 1*e *at center) - *E*(*N*), a quantity most related to symmetry, versus *Z *using *ε *= *ε*_0 _and known atomic radii and (b) measured **[30].

*Capacitance classically defined as charge per volt is no longer correct in QDs, not only quantum mechanically, but also classically, mainly due to Coulomb repulsion among the electrons in a typical QD*. When the number of electron becomes so large that they are pushed to the boundary, we reach the classical results that capacitance depends on geometry. We found that capacitance very much depends on number of electrons. We show results of *N*-electrons confined inside a dielectric sphere. A single electron is of course located at the center. With two, one pushed the other to the extremity of the boundary. For dielectric confinement, ε_in_> ε_out_, so that the induced charges at the boundary is of the same sign resulting in pushing the electron back from the boundary by its image, thereby achieving equilibrium. We calculated up to *N *= 108. Why? We basically obtained the periodic table of the chemical elements where all the elements are neutral. To compute the energy difference with *N *requires same number of charge as in atoms. Our computation of energy of interaction of *N*-electrons with that of *N *+ 1 electrons is based on minimization of the total interaction energy of the electrons without changing the charge state by adding an electron in the center without changing the overall symmetry. *Then the difference between N + 1 and N with one in the center is solely due to the change of symmetry*. Our results show that we have basically generated the periodic table. Figure 5 shows the actual positions up to 12 electrons. And Figure 6 shows the ionization energy quite comparable to the measured ionization energy. The point is about demonstrating the role of symmetry. The trend is as follows: adding an addition electron costs energy particularly adding an odd number, or worse yet, adding a prime number. In fact, I want to convince you that the most unique features of nanoscale physics are affected by the change of symmetry. *Therefore, conventional capacitance is only definable within a single phase, dictated by the unique symmetry. Measurements of capacitance are therefore related to exploring the symmetry*.

Due to complexity, the quantum mechanical computation was carried out only up to two electrons. I have been trying to find a student with strong computational skill to expand the QM computation to at least 12. Nevertheless, I can say something about. Capacitance is monophasic, i.e., each additional electron defines a single phase. And since the dielectric constant is much reduced in quantum mechanically confined systems, primarily because dielectric screening requires electrons or dipoles to move to cancel the applied electric field. Highly confined system reduces the movement, thereby reducing the screening. Now doping is basically possible because high dielectrically screened systems have very low binding energy, allowing carriers to be thermally excited at room temperatures. With drastic reduction of screening, the binding energy is too high, leading to carrier freeze-out at room temperatures. This is apart from the problem involving statistical factor due to the drastic reduction in size of the QDs. Doping is impossible.

### Summary of problems

In fact these problems discussed are serious, however, the most serious problem is I/O [14,20]. We reduce size to minimize real-estate. However, contacts are equal potentials, which call for metals. Nanosize metallic systems may be insolating, apart from the problem in lithography. Most of the bench-top demonstrations of Nanoelectronics have in-plane device configurations, not a real device. At this point I can conclude that with all the talk of nanoelectronics, the merit is perhaps due to special features, such as the THz devices, the QCL, and the new expectations in graphene-based electronics. It is true that MOSFET has been reduced to below 30 nm for the source-drain length, but there are still approximately 400 electrons in the channel-gate system, according to Ye [21]. Quantum computing is a somewhat unrealistic dream, because binary system makes computing possible, with the unique feature that on or off represents time-independent permanent states.

## Opportunities

### Quantum cascade laser with superlattice components

Quantum Cascade Laser was first succeeded at BTL under F. Capasso [5]. The idea was even patented before BTL succeeded. However, the patented version would not work because when many periods are in series, any fluctuation can start domain oscillation as pointed out by Gunn many years ago. Therefore, I shall single out QCL as an example how the problem is checked by introducing components each controlled separately as in QCL, with the three major components, the injector, the optical transition from the upper state to the lower state, and the collector. That is the direction of the superlattice, divided into components, together functioning as a device. With the exception of resistive switches, almost all devices such as MOSFET, flash memory, detectors, etc. involve components. In fact, the first optically pumped quantum well laser using very thin GaAs-AlGaAs QWs constitutes a step in the direction of utilizing QWs as components in forming a quantum device [22].

### THz sound in stark ladder superlattices

Application of an electric field to a weakly coupled semiconductor superlattice gives rise to an increase in the coherent folded phonon, generated by a femto-second optical pulse [23]. The condition is whenever the stark energy eFd > energy of the phonon, in this case, the FP phonon. Why did it take 35 years after the first article by Tsu and Döhler [24], to realize a phonon laser using superlattices? I want to make a comment from my years of doing research. Nobody is so brave in doing research in a relatively new field, although the instruments to fabricate devices involving superlattices are widely available. However, the complexity involved is sufficient in deterring most researchers. This study represents a step jump in the sophistication and careful design of the superlattice structure. I cannot fail to make a comment in regard to what Mark Reed told me about his study with pulling a gold wire while obtaining quantized conductance of the wire before it snapped. Some success is due to hard work, and others might be due to clever ideas and good timing. I would like to add from what happened today when Hashmi and I were jumping up and down for making a discovery. I said, "If you do something everybody else does, it is highly unlikely you would get anything new." The name of the game is to do something quite different!

### Cold cathode and graphene adventure

Cold cathode using resonant tunneling involving GaN [25] and using a layer of TiO_2 _[26] seem very different, but in fact are very similar, because both involve storing electrons in a region close to the surface by raising the Fermi level to effectively lowering the work function and resonantly tunneling out into the vacuum. Such schemes can be readily achieved when nanosized regions are created. And in a broad sense, creation of a region, or a component in general, as a section with electrons coherently related to the boundaries containing them.

**Figure 7:**
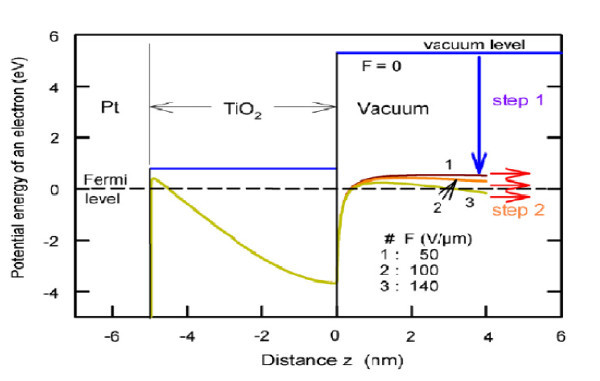
**Typical SSE with TiO_2 _on Pt**. Applied *E *field increases from 1: 50 V/μm, to 2: 100 V/μm, to 3: with 140 V/μm, showing increasing electron tunneling from *E_F_*, left, to the vacuum, right.

**Figure 8:**
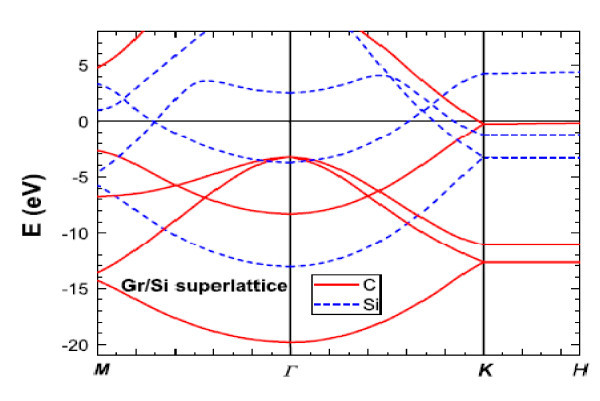
**Band structure of graphene/Si superlattice with *E_F _*= 0**. Solid and dashed are for the graphene and Si, respectively.*E_F _*is shifted above the linear dispersion at the k-point.

The graphene adventure took off more than anything I have seen in my entire life of research in solid state and semiconductors. In a way it reminded me of porous silicon because it involves silicon, the most widely used materials in electronic industry. However, the real reason is the availability of facilities to create porous silicon. All one needs is a kitchen sink. Ultimately it did not make the grade because porous silicon is not robust and mechanically stable. Using exfoliation, a little flake can represent a single layer of graphite allowing many to participate in this endeavor. However, I predict that unless controlled growth of graphene can be realized, the feverish activity will cease if large-scale growth of graphene cannot be realized. There is another major problem to be overcome. Graphene, a two-dimensional entity with *sp*^2 ^bonding configuration in reality does not exist, because we do not live in a two-dimensional world. And graphite consists of weak van der Waals bonding. Even in a single isolated layer, it is not graphene with only *sp*^2 ^bonds, because any real surface consists of surface reconstruction as well as adsorbents. And a stack of graphene forming graphite is best considered as lubricant, without mechanical stability and robustness. The answer lies in creating a graphene-based superlattice. Figure 7 shows a computed Graphene/Si superlattice using DFT [27] How to realize such a structure? Intercalation method would not work because it is hardly possible to introduce something uniform into the space between graphite planes. However, we know that nature creates coal with the Kaolin molecules, basically silicates and aluminates [28], in between the graphite layers. What represents in Figure 8 may very well be an empty wish, however, at this reporting, we are working toward growing Si/C superlattice.

### Some new opportunities

#### Beyond chemistry

As we know, chemistry deals with point group symmetry in the formation of molecules. When dealing with QDs, the boundary and shape of the QD provide extra symmetry relationships. Therefore, we are dealing with something new, which reminded me of the complexity of catalysts. Most catalysts have *d*-electrons, because the hybridization of *d*-*p *orbitals provides wide range of new possibilities to deal with symmetry configuration offered by the catalytic processes. I cannot help to imagine how a wide range of possibility opens up with nanosize QDs offering *new shapes *and *boundaries *to the wave functions. I for one am extremely interested in experiments enriching the understanding of the symmetry role in these quantum dots. *For example, we can use e-beam lithography to produce arrangements of dots representing various symmetry to study catalysis (nucleation in material growth)*. As we know that RPA, random phase approximation, introduced by Bohm and Pines as a catch phrase, no more than the recognition of not being able to take into account of phase relationship in totaling an interacting system. Most engineers would simply acknowledge the approximation by adding square-moduli to avoid cancellations. We do that in most constitutive relationships such as dielectric function, elastic constants, etc. We should be seriously considering the alternatives to adding square-moduli, or simply put, not using RPA. We know that the most powerful amplifier is the parametric amplifier where we cannot simply add oscillator strength. Ed Stern told me once why EXAFS is so powerful, because, with a giant computer, one can account for multiple scattering without resorting to the use of RPA, or nearest neighbor even next nearest neighbor interactions. As we pursue the nanoscience with ever increasing vigor using modern instruments such as AFM and STM having piezoelectric control of distance measured in nanometers, I think we should be seriously considering '*beyond RPA'*.

#### Beyond solid state physics

When we are working with a macroscopic entity, nature shows us the way-*translational symmetry*, normally referred to as solid state physics. As we know that nothing is perfect so that we resort to statistics to arrive at an average such as current, flow, etc. for the description of cause-effect as voltage-current, so useful for the description as well as the design of devices. As the size shrinks to dimensions in nanometers, the defects may be no more than zero or one in such way that statistical average does not apply. Many of the bench-top experiments I mentioned depends on what and where the device is, and whether we can control them or not. We can use statistics if there are many such devices in an en*semble average*, but not summing and averaging the individual scatterings! In simple term, translational symmetry does not play a part, and therefore, it is not solid state physics, but perhaps we should use the term *nano-solid*. Moreover, if the size is still represented by several unit cell distances, superlattice definitely is the only definable entity. In fact, even in the very first article [2], we pointed out that all one need is three periods in forming a superlattice, a QD in three-dimension.

#### Beyond composite

We shall go beyond electronics and optoelectronics to include the consideration of mechanical composites without glue. I envision a new kind of composite material consisting of components such as nanoscale entities dispersed in a matrix forming a composite instead of using nanorivets or glue, bonded together chemically as in superlattices, e.g., amorphous carbon as matrix, with embedded QDs of silicates. This recipe is not far from coal put together by nature.

#### Beyond biology

Superlattices have already broken into organic substances. It is only time to get involved with living organisms such as chlorophyll. Basically, now we have the tool to do it. I conclude here with one thought: Survival of the fittest for biological evolution should not be impeded by 'intelligent human technological advances' in nanoscience. However, we must be super-vigilant to avoid possible disasters to mankind.

## Abbreviations

ATM: atomic force microscopy; MBE: molecular beam epitaxy; NDC: negative differential conductance; QDs: quantum dots; QWS: quantum wells; RPA: random phase approximation; STM: scanning tunneling microscopy.

## Competing interests

The author declares that they have no competing interests.
